# Atelectasis predicts poor prognosis in pediatric macrolides-unresponsive *Mycoplasma pneumoniae* pneumonia with A2063/2064G mutations treated with azithromycin

**DOI:** 10.3389/fcimb.2025.1604102

**Published:** 2025-06-27

**Authors:** Jie Cheng, Ya Liu, Guangli Zhang, Yuanyuan Li, Xiaoyin Tian, Liping Tan, Zhengxiu Luo

**Affiliations:** ^1^ Department of Emergency, National Clinical Research Center for Child Health and Disorders, Ministry of Education Key Laboratory of Child Development and Disorders, Chongqing Key Laboratory of Child Rare Diseases in Infection and Immunity, Intelligent Application of Big Data in Pediatrics Engineering Research Center of Chongqing Education Commission of China, Children’s Hospital of Chongqing Medical University, Chongqing, China; ^2^ Department of Pediatrics, Chongqing Youyoubaobei Women and Children’s Hospital, Chongqing, China; ^3^ Department of Respiratory Medicine, National Clinical Research Center for Child Health and Disorders, Ministry of Education Key Laboratory of Child Development and Disorders, Chongqing Key Laboratory of Child Rare Diseases in Infection and Immunity, Intelligent Application of Big Data in Pediatrics Engineering Research Center of Chongqing Education Commission of China, Children’s Hospital of Chongqing Medical University, Chongqing, China

**Keywords:** atelectasis, *Mycoplasma pneumoniae* pneumonia, A2063/2064G mutation, azithromycin, children

## Abstract

**Objective:**

We aimed to investigate prognostic indicators for pediatric macrolides-unresponsive *Mycoplasma pneumoniae* pneumonia (MUMPP) cases with A2063/2064G mutations with azithromycin therapy.

**Methods:**

This was a retrospective observational cohort study conducted at the Children’s Hospital of Chongqing Medical University. Children with macrolide-resistant mutations (A2063/2064G) diagnosed as MUMPP who received only anti-*Mycoplasma pneumoniae* (MP) treatment with azithromycin were retrospectively enrolled. Logistic regression analysis was used to identify potential risk factors for predicting short-term (refractory *Mycoplasma pneumoniae* pneumonia [RMPP]) and long-term (bronchiolitis obliterans [BO] or bronchiectasis) adverse prognosis. The results were visualized using forest plots.

**Results:**

This study retrospectively included 82 children with MUMPP, and all received only azithromycin for anti-MP treatment. The incidence of pulmonary consolidation, pleural effusion, and atelectasis was 80.49% (66/82), 34.15% (28/82), and 24.39% (20/82), respectively. 29.27% (24/82) of patients diagnosed with RMPP, and 14.63% (12/82) of patients diagnosed with bronchiolitis obliterans (BO) or bronchiectasis diagnosed within one year after discharge. Logistic analysis showed that atelectasis was independently associated with short-term (RMPP) and long-term (BO or bronchiectasis) adverse prognosis (odds ratio [OR] 4.02, 95% confidence interval [CI] 1.03-16.00, P = 0.043; OR 5.62, 95% CI 1.04-32.80, P = 0.045; respectively).

**Conclusion:**

Atelectasis predicts a poor prognosis for children with A2063/2064G MUMPP. The occurrence of atelectasis may indicate an increased risk of failure of current azithromycin treatment. Combined with the results of drug-resistant mutations, it is recommended to strengthen disease monitoring and individualized intervention evaluation.

## Introduction

*Mycoplasma pneumoniae* (MP) is one of the most common respiratory tract infection pathogens in children, accounting for 20%-40% of community-acquired pneumonia in children (Wang et al., 2024). Most of the time, it is self-limiting. However, some children have poor prognosis after infection, such as macrolide-unresponsive *Mycoplasma pneumoniae* pneumonia (MUMPP) (National Health Commission of the People’s Republic of China, 2024), refractory *Mycoplasma pneumoniae* pneumonia (RMPP) (National Health Commission of the People’s Republic of China, 2024), bronchiolitis obliterans (BO) (Zhang et al., 2023), bronchiectasis (Guo et al., 2024), etc. All the poor prognosis may be related to the drug resistance of MP (Zhang et al., 2023) and the MP-induced immune dysregulation (Zhang et al., 2023; Guo et al., 2024). The problem of MP resistance to macrolides is becoming increasingly prominent (Zhang et al., 2023). It is reported that the resistance rate of MP to macrolide drugs has reached more than 70% (Kim et al., 2022). In China, the resistance rate to azithromycin after MP infection in adults can be as high as 100% (Pereyre et al., 2016). These are thought to be associated with exposure of MP to azithromycin and the occurrence of the A2063/2064G gene mutation (Oishi et al., 2024). However, due to the unique characteristics of children, the safety of tetracyclines and quinolones in children needs further consideration (Cai et al., 2024), and azithromycin is still the first-line drug of choice for the treatment of *Mycoplasma pneumoniae* pneumonia (MPP) in children (Gao and Sun, 2024). The increasing resistance of MP to azithromycin and the good efficacy of tetracyclines and quinolones against MP infection (Cai et al., 2024) make it difficult for pediatricians to choose between efficacy and safety. The A2063/2064G gene mutation is the leading cause of macrolide resistance in MP (Lucier et al., 1995). However, previous studies have found that azithromycin still has good clinical efficacy in some children with MPP with A2063/2064G gene mutations (Cheng et al., 2024). In addition, azithromycin combined with glucocorticoids or immunoglobulin is effective in children with RMPP (Wang et al., 2024). MUMPP refers to pediatric MPP with persistent fever after 72 hours of regular treatment with macrolide antibiotics, without improvement of clinical signs or lung imaging, which is generally considered ineffective in treatment with azithromycin alone (National Health Commission of the People’s Republic of China, 2024). However, clinicians must carefully consider choosing azithromycin combined with glucocorticoids or immunoglobulin or switching to tetracyclines or quinolones for children with MUMPP. Furthermore, predictors of treatment failure in this subgroup of patients remain unclear.

Considering all the above, we aimed to identify prognostic markers for pediatric MUMPP cases with A2063/2064G mutations treated with azithromycin monotherapy, prompting clinicians to consider alternative therapies and improving outcomes.

## Methods

### Study design and population

This single-center, retrospective, observational cohort study was conducted at the Children’s Hospital of Chongqing Medical University. From January 2019 to December 2022, hospitalized children diagnosed with MPP and A2063/2064G gene mutation were retrospectively included in the study. All outpatient or inpatient data of all patients one year after discharge were collected. Inclusion criteria were as follows: (i) hospitalized children and (ii) diagnosed with macrolide resistance mutation (A2063/2064G)-related MPP. Exclusion criteria were as follows: (i) patients with defervescence before admission; (ii) patients with incomplete clinical data; (ii) patients with hematological malignancies; (iv) patients did not diagnosed with MUMPP, and (v) patients who had received tetracyclines or quinolones during hospitalization. This study protocol was approved by the Ethics Committee of the Children’s Hospital Affiliated to Chongqing Medical University (approval number: 2021-179), and the patient’s informed consent was explicitly waived. This waiver was in accordance with the hospital’s retrospective research policy and the ethical guidelines of the Declaration of Helsinki (1964 and subsequent revisions). This study did not involve clinical trials or animal experiments, and all data processing strictly complied with the above ethical standards.

### Data collection and definitions

We retrospectively obtained information including demographic data, underlying diseases, laboratory data, chest imaging data, antibiotic treatment during hospitalization, fever duration, cough duration, diagnosis, and prognosis. The *Mycoplasma pneumoniae* pneumonia (MPP), macrolide-unresponsive *Mycoplasma pneumoniae* pneumonia (MUMPP), and refractory *Mycoplasma pneumoniae* pneumonia (RMPP) were diagnosed according to the Chinese Guidelines for Diagnosis and Treatment of Mycoplasma Pneumoniae Pneumonia in Children (2023 Edition) (National Health Commission of the People’s Republic of China, 2024). Bronchiolitis obliterans (BO) (Zhang et al., 2023) and bronchiectasis (Peroni and Boner, 2000) were mainly diagnosed through chest imaging and bronchoscopy combined with clinical symptoms and signs. According to the Peroni criteria (Peroni and Boner, 2000), atelectasis is defined as decreased lung volumes with loss of air bronchograms, whereas consolidation is defined as alveolar filling with exudate but preserved volume. The chest X-ray anteroposterior and lateral projections were used as the preferred method for diagnosing atelectasis (which met the conventional diagnostic standards), and chest computed tomography (CT) was performed to improve the resolution in difficult cases (Peroni and Boner, 2000). Image evaluation was performed independently by two radiologists who were unaware of clinical information (double-blind design). When the results were inconsistent, senior physicians other than the first two made a decision after discussion to minimize diagnostic bias. All children implemented a structured follow-up plan: the first visit to the respiratory specialist clinic 1–2 weeks after discharge was for a comprehensive assessment based on symptoms/signs; if there was persistent cough, wheezing, or abnormal oxygenation, a chest CT or bronchoscopy was triggered. Asymptomatic patients were not routinely reviewed with imaging. All cases completed a minimum of 1 year of follow-up, and endpoint events (BO/bronchiectasis) were confirmed by imaging.

### Macrolide resistance gene analysis

Clinical specimens including bronchoalveolar lavage fluid (BALF) and nasopharyngeal aspirate (NPA) were evaluated for mutations at the A2063G/A2064G locus. Genetic resistance analysis was performed by nested polymerase chain reaction (nPCR), capillary electrophoresis, and single-strand conformation polymorphism (CE-SSCP) according to established molecular testing protocols (Lin et al., 2010).

### Clinical outcomes

The short-term outcome was the occurrence of RMPP, and the long-term outcome was the diagnosis of BO or bronchiectasis.

### Statistical analysis

The distribution of continuous data was assessed using the Mann-Whitney U test or Student’s t test, and the results are expressed as median (interquartile range [IQR]). Categorical comparisons were performed using Pearson χ^2^ or Fisher’s exact test, and frequency counts were expressed as proportions (%). Logistic regression analysis was used to identify potential risk factors for predicting adverse prognosis in the short-term (occurrence of RMPP) and long-term (diagnosis of BO or bronchiectasis). Predictors with P ≥ 0.10 in the initial univariate screening were retained for subsequent multivariate modeling. The area under the ROC curve (AUC) is used to evaluate the predictive performance of the logistic regression model: when the AUC value is between 0.5 and 0.7, it indicates limited predictive ability, 0.7 to 0.9 indicates moderate predictive ability, and above 0.9 indicates excellent predictive ability (Cheng et al., 2020). The final regression results were evaluated for prognostic efficacy using odds ratios (ORs) and 95% confidence intervals (CIs). And the results were visualized using forest plots. P value < 0.05 was considered statistically significant (two-sided). All analyses were performed using R software version 4.3.2.

## Results

### Study population

**Figure 1:**
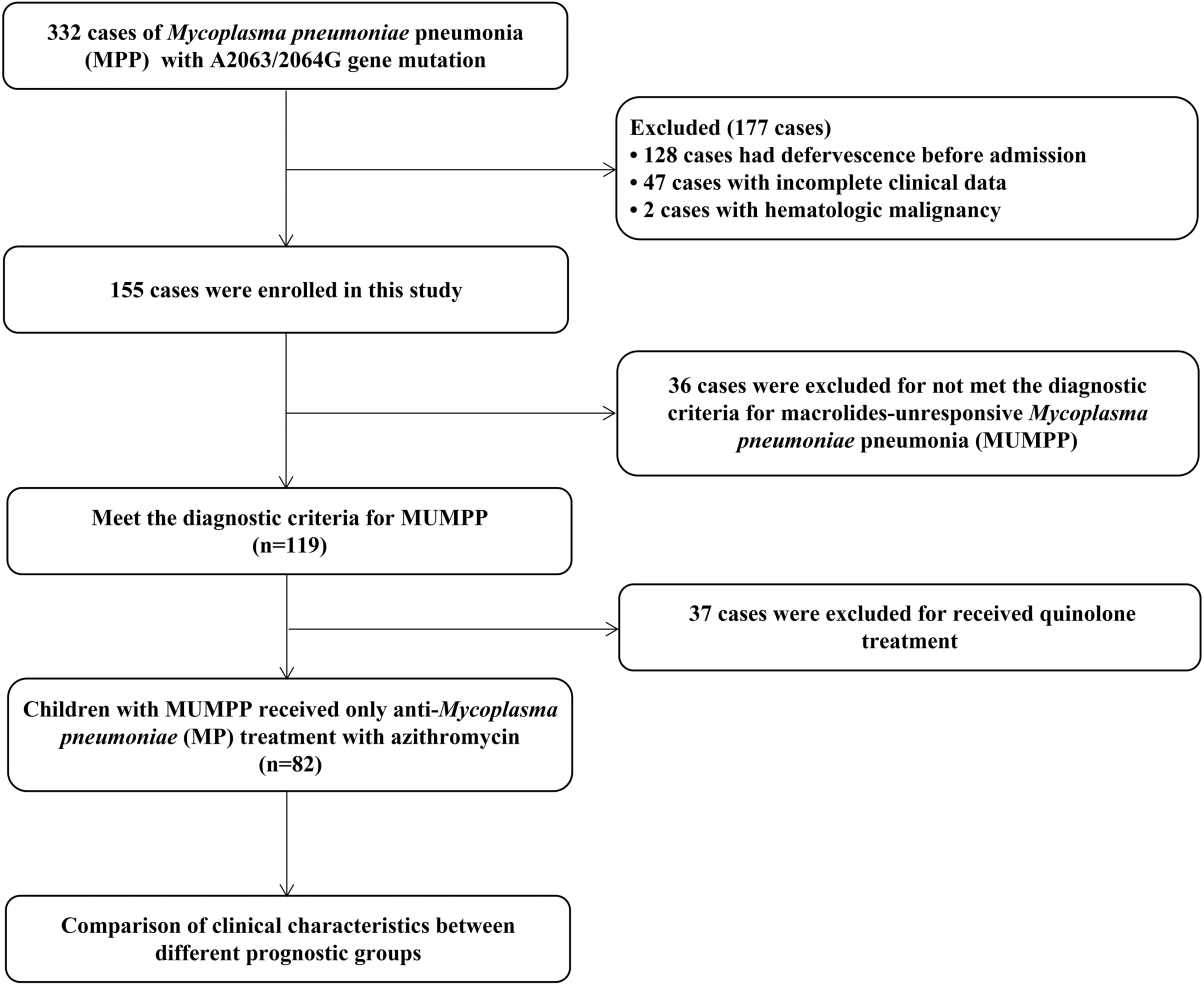
Flowchart of this study.

During the study period 2019-2022, three hundred and thirty-two pediatric cases initially met inclusion criteria. Exclusions (n = 250) included defervescence before admission (n = 128), incomplete records (n = 47), received tetracyclines or quinolones during hospitalization (n = 37), diagnosed with MUMPP (n = 36) or hematological malignancies (n = 2), resulting in a total of 82 evaluable participants. The flow chart of this study is shown in **Figure 1**.

### Clinical characteristics of 82 MUMPP children with A2063/2064G gene mutation who received only anti-MP treatment with azithromycin

The median age was 6.20 (IQR 4.19-8.15) years, and the median length of hospital stay was 13.75 (IQR 11.33-17.59) days. The median fever course before anti-MP therapy was 5.00 (IQR 3.00-6.00) days, and the total fever course was 10.00 (IQR 9.00-13.00) days. There were 16 (16/82, 19.51%) children with a concurrent viral infection, and 6 (6/82, 7.32%) children with a concurrent bacterial infection. The incidence of pulmonary consolidation, pleural effusion, and atelectasis was 80.49% (66/82), 34.15% (28/82), and 24.39% (20/82), respectively. There were 5 (5/82, 6.10%) patients who received interrupted/reinitiated azithromycin therapy. The proportion of patients using glucocorticoids and immunoglobulin was 35.27% and 14.63% respectively. More than two-thirds (73.17%, 60/82) of patients underwent bronchoscopy, and less than 10% (8.54%, 7/82) required non-invasive ventilator support. More than 30% (31.71%, 26/82) of children are readmitted to the hospital within a month due to MPP. At last, 29.27% (24/82) of patients diagnosed with RMPP and 14.63% (12/82) of patients diagnosed with bronchiolitis obliterans (BO) or bronchiectasis within one year after discharge. See **Table 1** for detailed data.

**Table 1:** Characteristics of 82 macrolides-unresponsive *Mycoplasma pneumoniae pneumonia* (MUMPP) children with gene A2063/2064G mutation who received only anti-*Mycoplasma pneumoniae* (MP) treatment with azithromycin.

Characteristics	Number (%)/Median (IQR)
Demographic data	
Age (years) (median, IQR)	6.20 (4.19-8.15)
Weight (kg) (median, IQR)	20.70 (16.00-25.23)
Male (n, %)	41 (50.00%)
Laboratory tests	
White blood cell (WBC) level (10^9/L) (median, IQR)	7.04 (4.84-9.55)
Neutrophil level (10^9/L) (median, IQR)	67.64 (60.17-73.77)
C-reactive protein (CRP) level (mg/L) (median, IQR)	19.51 (11.30-38.28)
Procalcitonin (PCT) level (ng/ml) (median, IQR)	0.21 (0.11-0.53)
Fibrinogen level (g/L) (median, IQR)	4.54 (4.06-4.90)
D-dimer level (ng/ml) (median, IQR)	0.83 (0.45-2.08)
Serum albumin level (g/L) (median, IQR)	40.30 (36.03-43.45)
Alanine transaminase (ALT) level (U/L) (median, IQR)	16.00 (13.00-22.00)
Aspartate aminotransferase (AST) level (U/L) (median, IQR)	35.50 (28.00-44.00)
Lactate dehydrogenase (LDH) (U/L) level (median, IQR)	325.50 (278.75-442.08)
Imaging findings	
Consolidation (n, %)	66 (80.49%)
Pleural effusion (n, %)	28 (34.15%)
Atelectasis (n, %)	20 (24.39%)
Co-infection pathogen	
Virus (n, %)	16 (19.51%)
Bacteria (n, %)	6 (7.32%)
Medication regimen	
Intravenous penicillin or cephalosporins (n, %)	39 (47.56%)
Intravenous corticosteroids (n, %)	29 (35.37%)
Intravenous immunoglobulin (n, %)	12 (14.63%)
With bronchoscopy (n, %)	60 (73.17%)
Non-invasive ventilator support required (n, %)	7 (8.54%)
With interrupted/reinitiated azithromycin therapy (n, %)	5 (6.10%)
The highest body temperature during MPP infection (°C) (median, IQR)	40.0 (39.5-40.2)
Fever course before anti-MP therapy (days) (median, IQR)	5.00 (3.00-6.00)
Total fever course (days) (median, IQR)	10.00 (9.00-13.00)
Length of hospital stay (days) (median, IQR)	13.75 (11.33-17.59)
Readmission due to MPP within 1 month (n, %)	26 (31.71%)
Outcomes	
Refractory *Mycoplasma pneumoniae* pneumonia (RMPP) (n, %)	24 (29.27%)
Bronchiolitis obliterans (BO) or bronchiectasis diagnosed within one year after discharge (n, %)	12 (14.63%)

### Clinical characteristics comparisons of 82 children with A2063/2064G gene mutation who received only anti-MP treatment with azithromycin in the RMPP group and the non-RMPP

The neutrophil level, C-reactive protein (CRP) level, D-dimer level, and lactate dehydrogenase (LDH) level of the RMPP group were significantly higher than those of the non-RMPP group (P <0.05). The total duration of fever in the RMPP group was also significantly longer than that in the non-RMPP group (13.00 days (IQR 10.75-14.25 days) *vs* 10.00 days (IQR 9.00-11.00 days), P <0.001). There were significantly more patients in the RMPP group who had atelectasis (54.17% *vs*. 12.07%, P <0.001), received glucocorticoids (70.83% *vs*. 20.69%, P <0.001), and were readmitted for MPP within 1 month (62.50% *vs*. 18.97%, P <0.001) compared with the non-RMPP group. The demographic data (age, weight, sex), the highest body temperature during MPP infection, total length of hospital stay, white blood cell (WBC) level procalcitonin (PCT) level, fibrinogen level, D-dimer level, serum albumin level, alanine transaminase (ALT) level, and aspartate aminotransferase (AST) level were with no statistically significant difference. In addition, there was no significant statistical difference between the RMPP and the non-RMPP groups in the number of patients using glucocorticoids, the number of patients requiring non-invasive ventilator support, and the number of patients receiving immunoglobulin therapy. For details, see **Table 2**.

**Table 2:** Clinical characteristics comparisons of 82 children with A2063/2064G gene mutation who received only anti-MP treatment with azithromycin in the RMPP group and the non-RMPP group.

Characteristics	Non-RMPP (n=58)	RMPP (n=24)	P value
Demographic data			
Age (years) (median, IQR)	6.55 (4.69-8.23)	5.50 (3.88-7.52)	0.504
Weight (kg) (median, IQR)	20.20 (16.35-25.23)	21.00 (15.00-25.25)	0.870
Male (n, %)	31 (53.45%)	10 (41.67%)	0.467
Laboratory tests			
WBC level (10^9/L) (median, IQR)	7.07 (5.08-9.69)	6.61 (4.58-8.48)	0.460
Neutrophil level (10^9/L) (median, IQR)	65.68 (58.87-73.05)	70.52 (65.20-75.27)	0.042*
CRP level (mg/L) (median, IQR)	16.96 (10.67-29.60)	35.00 (14.75-54.00)	0.010*
PCT level (ng/ml) (median, IQR)	0.18 (0.10-0.42)	0.41 (0.11-0.60)	0.143
Fibrinogen level (g/L) (median, IQR)	4.54 (3.96-4.92)	4.67 (4.16-4.79)	0.870
D-dimer level (ng/ml) (median, IQR)	0.61 (0.41-1.13)	2.25 (0.81-4.41)	<0.001*
Serum albumin level (g/L) (median, IQR)	41.60 (39.08-43.78)	36.50 (32.85-42.00)	0.002*
ALT level (U/L) (median, IQR)	15.00 (13.00-20.75)	17.90 (3.00-28.53)	0.238
AST level (U/L) (median, IQR)	34.50 (28.00-42.93)	36.00 (26.73-44.53)	0.748
LDH level (U/L) (median, IQR)	307.00 (266.50-364.00)	448.55 (315.50-641.00)	0.002*
Imaging findings			
Consolidation (n, %)	44 (75.86%)	22 (91.67%)	0.131
Pleural effusion (n, %)	16 (27.59%)	12 (50.00%)	0.091
Atelectasis (n, %)	7 (12.07%)	13 (54.17%)	<0.001*
Co-infection pathogen			
Virus (n, %)	12 (20.69%)	4 (16.67%)	0.768
Bacteria (n, %)	4 (6.90%)	2 (8.33%)	1.000
Medication regimen			
Intravenous penicillin or cephalosporins (n, %)	23 (39.66%)	16 (66.67%)	0.047
Intravenous corticosteroids (n, %)	12 (20.69%)	17 (70.83%)	<0.001*
Intravenous immunoglobulin (n, %)	9 (15.52%)	3 (12.50%)	1.000
With bronchoscopy (n, %)	39 (67.24%)	21 (87.50%)	0.107
Non-invasive ventilator support required (n, %)	3 (5.17%)	4 (16.67%)	0.186
With interrupted/reinitiated azithromycin therapy (n, %)	5 (8.62%)	0 (0.00%)	0.315
The highest body temperature during MPP infection (°C) (median, IQR)	40.0 (39.5-40.3)	40.0 (39.6-40.0)	0.737
Fever course before anti-MP therapy (days) (median, IQR)	5.00 (3.00-6.00)	5.00 (3.00-7.00)	0.881
Total fever course (days) (median, IQR)	10.00 (9.00-11.00)	13.00 (10.75-14.25)	<0.001*
Length of hospital stay (days) (median, IQR)	13.96 (11.37-17.29)	13.71 (11.38-18.02)	0.887
Readmission due to MPP within 1 month (n, %)	11 (18.97%)	15 (62.50%)	<0.001*
BO or bronchiectasis diagnosed within one year after discharge (n, %)	2 (3.45%)	10 (41.67%)	<0.001*

*with statistical significance, P <0.05.

### Logistic regression analysis for risk factors of short-term poor prognosis in 82 children with A2063/2064G gene mutation who received only anti-MP treatment with azithromycin

**Figure 2:**
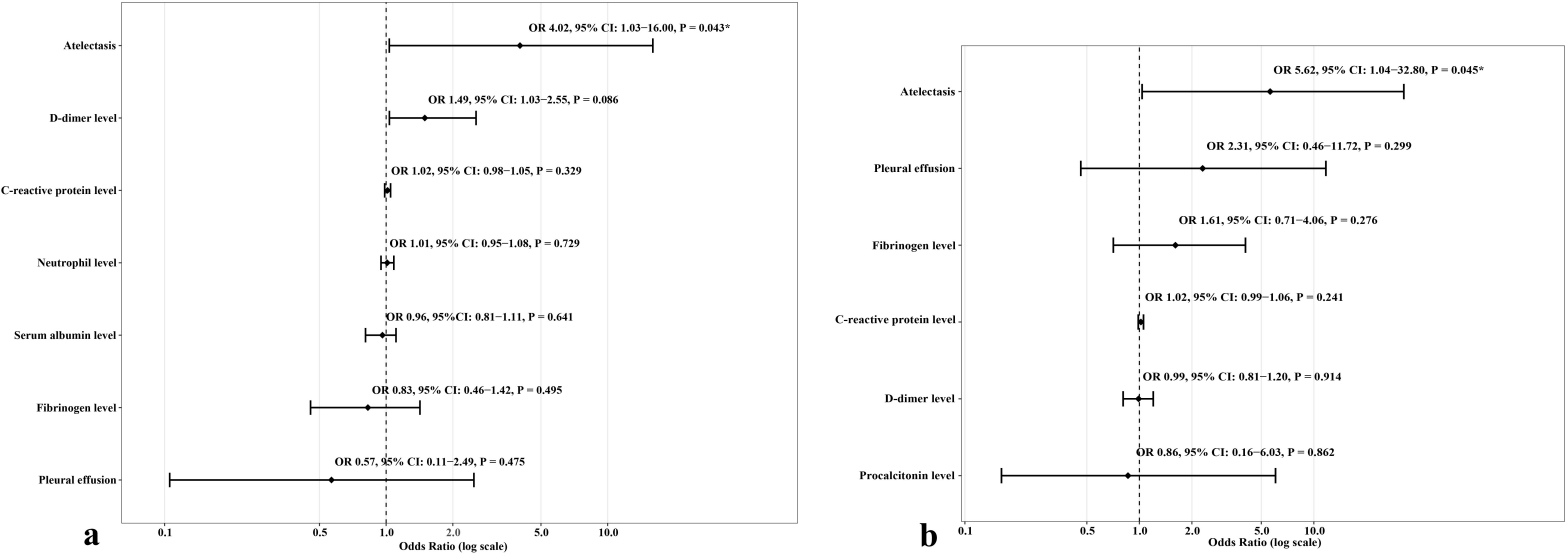
Forest plots presenting odds ratios (OR) and 95% confidence intervals (CI) for refractory *Mycoplasma pneumoniae* pneumonia (RMPP) **(a)** and long-term poor prognosis (bronchiolitis obliterans or bronchiectasis) **(b)**, stratified by atelectasis.

In univariate logistic analysis, atelectasis, pleural effusion, high neutrophil level, high CRP level, high D-dimer level, high LDH level, and low serum albumin level were remarkably associated with the risk of developing RMPP. In multivariate analysis, atelectasis was the only independent risk factor of RMPP (odds ratio [OR] 4.02, 95% confidence interval [CI] 1.03-16.00, P = 0.043). The AUC of atelectasis in predicting short-term poor prognosis (RMPP) was 0.75 (95% CI 0.60-0.89), indicating moderate predictive efficacy. Details are shown in **Table 3**; **Figure 2a**. More importantly, after re-analyzing the multivariate model with age and consolidation included as covariates, atelectasis remained a significant independent predictor (details are shown in **Supplementary Table S1**).

**Table 3:** Logistic regression analysis for risk factors of short-term poor prognosis (RMPP) in 82 children with A2063/2064G gene mutation who received only anti-MP treatment with azithromycin.

Variables	Univariate analysis			Multivariate analysis		
	OR	95% CI	P	OR	95% CI	P
Atelectasis (n, %)	8.61	2.89-28.04	<0.001*	4.02	1.03-16.00	0.043*
Pleural effusion (n, %)	2.63	0.98-7.14	0.055			
D-dimer level (ng/ml) (median, IQR)	1.83	1.30-2.86	0.003*			
CRP level (mg/L) (median, IQR)	1.03	1.01-1.06	0.006*			
LDH level (U/L) (median, IQR)	1.01	1.00-1.01	<0.001*			
Neutrophil level (10^9/L) (median, IQR)	1.01	1.00-1.09	0.050			
Serum albumin level (g/L) (median, IQR)	0.84	0.75-0.93	0.002*			

*with statistical significance, P <0.05.

### Logistic regression analysis for risk factors of long-term poor prognosis (BO or bronchiectasis diagnosed within one year after discharge) in 82 children with A2063/2064G gene mutation who received only anti-MP treatment with azithromycin

Univariate regression analysis showed that atelectasis, pleural effusion, high PCT level, high fibrinogen level, high D-dimer level, and high CRP level were significantly associated with the occurrence of long-term adverse prognosis (BO or bronchiectasis diagnosed within one year after discharge). The multivariate logistic analysis showed that atelectasis was independently associated with the occurrence of long-term adverse prognosis (OR 5.62, 95% CI 1.04-32.80, P = 0.045). The AUC of atelectasis in predicting long-term poor prognosis (BO or bronchiectasis diagnosed within one year after discharge) was 0.71 (95% CI 0.60-0.82), indicating moderate predictive efficacy. Details are shown in **Table 4**; **Figure 2b**. Furthermore, after re-analyzing the multivariate model with age and consolidation included as covariates, atelectasis remained a significant independent predictor (details are shown in **Supplementary Table S2**).

**Table 4:** Logistic regression analysis for risk factors of long-term poor prognosis (BO or bronchiectasis diagnosed within one year after discharge) in 82 children with A2063/2064G gene mutation who received only anti-MP treatment with azithromycin.

Variables	Univariate analysis			Multivariate analysis		
	OR	95% CI	P	OR	95% CI	P
Atelectasis (n, %)	9.67	2.62-41.39	0.001*	5.62	1.04-32.80	0.045*
Pleural effusion (n, %)	5.00	1.41-20.52	0.016*			
PCT level (ng/ml) (median, IQR)	3.93	1.21-23.23	0.084			
Fibrinogen level (g/L) (median, IQR)	1.88	1.05-3.95	0.050			
D-dimer level (ng/ml) (median, IQR)	1.17	1.00-1.38	0.047*			
CRP level (mg/L) (median, IQR)	1.04	1.01-1.07	0.008*			

*with statistical significance, P <0.05.

## Discussion

MP has attracted much attention from clinicians as one of the most common pathogens of community-acquired pneumonia in children. In recent years, macrolide resistance in MP is increasingly prevalent. The resistance rate in adults has reached more than 70% (Kim et al., 2022), and in China, the resistance rate is even as high as 100% (Pereyre et al., 2016). However, given the unique characteristics of children and the safety of medication, azithromycin is still the first-line drug for the treatment of MP. Furthermore, azithromycin still has good clinical efficacy in some children with MPP with A2063/2064G gene mutations (Cheng et al., 2024), considered the leading cause of MP resistance to macrolides (Lucier et al., 1995). Although the effect of switching to tetracyclines or quinolones for children with MUMPP is considerable, the clinical efficacy of azithromycin combined with immunoglobulin or glucocorticoids is also considerable. It can avoid the safety uncertainty of tetracyclines or quinolones. Then, our study investigated the risk factors for short-term (RMPP) and long-term (BO or bronchiectasis) adverse outcomes in children with MUMPP with A2063/2064G gene mutation treated with azithromycin (without tetracycline or quinolones). To our knowledge, this is the first study to identify atelectasis as a prognostic marker, specifically in pediatric MUMPP cases with A2063/2064G mutations treated with azithromycin monotherapy.

Atelectasis is a condition in which one or more areas of the lung collapse and fail to inflate normally due to a decrease or complete loss of air in the alveoli (Peroni and Boner, 2000), which can lead to alveolar collapse and ventilation dysfunction, causing an imbalance in the local ventilation-blood flow ratio, affecting oxygenation function, and in severe cases may cause hypoxemia or respiratory failure (Peroni and Boner, 2000). In this study, we found that atelectasis was an independent risk factor of RMPP (OR 4.02, 95% CI 1.03-16.00, P = 0.043). Although the A2063/2064G gene mutation may play an important role in the development of RMPP in children with MPP, Deng et al (Deng et al., 2018). found that the A2063/2064G gene mutation of MP is not associated with chest imaging findings in children with pneumonia. Then, it is reasonable to apply atelectasis as a predictive indicator for RMPP in children with MPP with A2063/2064G gene mutation. The possible mechanism of atelectasis as an indicator of RMPP is as follows. First, MP infection promotes inflammatory response and eliminates pathogens through communication between alveolar epithelial cells and alveolar macrophages (Xue et al., 2023). Alveolar macrophages play a crucial role in maintaining lung homeostasis and protecting alveolar epithelial function and integrity by increasing the production of inflammatory mediators (Baloglu et al., 2022). Atelectasis causes local hypoxia in the lung tissue, increases the permeability of the alveolar barrier, and activates alveolar macrophages to release inflammatory mediators, which leads to edema in the lung tissue. Pulmonary edema can further worsen pulmonary hypoxia, while inflammation and hypoxia may further aggravate pulmonary edema and damage, creating a vicious cycle (Chao et al., 2009). This persistent excessive inflammation may lead to persistent fever, aggravating lung imaging and clinical symptoms, and prompting of RMPP in children with MPP after macrolide treatment (Lee et al., 2021). Second, clinical studies have found that atelectasis affects the penetration of drugs into affected lung tissue (Hutschala et al., 2008). Atelectasis leads to decreased blood flow and mucus accumulation in the affected area (Peroni and Boner, 2000), which may also result in significantly reduced local concentrations of antibiotics (such as macrolides), and lead to treatment failure. Last, the affected lung tissue of atelectasis has a reduced ability to clear pathogens (Drinkwater et al., 1981), leading to persistent MP infection and even progression to RMPP. However, it is worth noting that atelectasis, as an early imaging event (earlier than immunotherapy intervention), may amplify local inflammatory responses by aggravating ventilation/perfusion imbalance, prompting clinical escalation of treatment (such as glucocorticoids or immunoglobulins). This temporal independence strengthens its warning value, but the net effect of immunomodulatory therapy on prognosis still needs to be clarified through randomized controlled designs. Furthermore, atelectasis is a warning marker, and the benefit of intervention needs to be prospectively verified. Although a higher proportion of children with atelectasis received bronchoscopy (90.0% *vs* 67.7%, P=0.096), there was no difference in the risk of mechanical ventilation between children with atelectasis and those without atelectasis in the conservative treatment subgroup (0.00% *vs* 0.00%, P=1.000). Combined with the fact that there was no difference in the use of CPAP between the two groups (10.0% *vs* 8.06%, P=1.000), it suggests that atelectasis is more of an imaging marker of the severity of the underlying disease rather than an intervention target that directly causes respiratory failure. In the future, randomized controlled trials are needed to clarify the net benefit of bronchoscopy for children with atelectasis.

In this study, we also found that among children with MUMPP who received azithromycin monotherapy (without tetracyclines or quinolones) and with A2063/2064G gene mutation, those with atelectasis had a 5.62-fold increased risk of developing BO or bronchiectasis within one year compared with those without atelectasis (OR 5.62, 95% CI 1.04-32.80, P = 0.045). The possible mechanism is as follows. First, atelectasis-related mucus plugs can activate NLRP3 inflammasomes (Tran et al., 2020), promote the release of proinflammatory cytokines such as IL-1β, and aggravate peribronchiolar fibrosis (Nguyen et al., 2024). These may lead to irreversible airway remodeling, leading to BO or bronchiectasis. Second, atelectasis leads to obstruction of bronchial drainage, mucus plugs, and pathogens accumulate locally (Peroni and Boner, 2000), and the affected lung tissue of atelectasis has a reduced ability to clear pathogens (Drinkwater et al., 1981). This leads to recurrent infections, further damaging the bronchial wall’s elastic fibers and smooth muscles, weakening the airway’s self-cleaning ability, and forming a vicious cycle of “atelectasis - infection - expansion”. Last, atelectasis can lead to alveolar hypoxia and chronic inflammation (Tojo et al., 2015), and this hypoxia and chronic inflammation may lead to bronchial fibrotic remodeling (Racanelli et al., 2018) and may cause bronchial stenosis or dilation.

This study found that atelectasis significantly increased the risk of adverse prognosis in children with A2063/2064G mutation MUMPP (RMPP: OR 4.02, 95% CI 1.03-16.00; BO/bronchiectasis: OR 5.62, 95% CI 1.04-32.80). Accordingly, we proposed a clinical management protocol in combination with the study of Peroni et al (Peroni and Boner, 2000). (**Supplementary Figure S1**): First, all children with A2063/2064G mutation MUMPP need to undergo initial chest X-ray for atelectasis detection, with escalation to chest CT if results are inconclusive. Second, for those who are diagnosed with atelectasis, intensive treatment (physical expectoration plus mucolytic drugs) will be initiated immediately. If there is no improvement in clinical symptoms or imaging, bronchoscopy, antibiotic escalation (e.g., tetracyclines for ≥8 years), and immunomodulation (corticosteroids/immunoglobulin) should be evaluated. Last, long-term follow-up focuses on monitoring bronchiectasis/BO (CT reexamination is recommended in 1/3/6 months). This protocol aims to improve the prognosis of children with drug-resistant mutations through early intervention.

There are some limitations. First, this study is a small sample, single-center study, the small sample size (n=82) resulted in insufficient statistical power, which was manifested in the wide confidence interval of the OR value for the association between atelectasis and prognosis (1.03-16.00 in the short term and 1.04-32.80 in the long term). Although the specific effect values need to be interpreted with caution, the significance of atelectasis as an independent predictor (P <0.05) and its high OR value (>4) still suggest its clinical warning value. In the future, multicenter large-sample studies are needed to further verify the strength of this association. Second, this is a retrospective study. Reliance on historical records can easily lead to incomplete control of confounding factors (such as treatment differences and unmeasured variables) and lack of prospective verification and long-term follow-up, affecting causal inference’s reliability. In the future, multicenter prospective studies are expected to verify the prognostic value of atelectasis, and further explore the pathological mechanism of atelectasis through inflammatory factor spectrum analysis of bronchoalveolar lavage fluid to promote the optimization of diagnosis and treatment strategies. Third, this study included only children and may not reflect the pathological differences in adults. Caution is needed when extrapolating conclusions to all age groups. Last, in order to clarify the efficacy of azithromycin alone, only children who did not respond to azithromycin anti-mycoplasma treatment for ≥ 72 hours were included, and those who used tetracycline/quinolones were actively excluded. This resulted in the exclusion of critically ill children who needed to switch to tetracycline/quinolone drugs (which may underestimate the severity of the included population). However, this design allows us to focus on the prognostic value of atelectasis in patients who have failed azithromycin treatment in the context of macrolide resistance mutations, providing a specific reference for similar resistant cases. Future prospective studies are needed to compare the prognostic differences of different antibiotic strategies.

## Conclusion

This study found that atelectasis serves as a prognostic marker in pediatric MUMPP cases with A2063/2064G mutations treated with azithromycin monotherapy. The occurrence of atelectasis may indicate an increased risk of failure of current azithromycin treatment. Combined with the results of drug-resistant mutations, it is recommended to strengthen disease monitoring and individualized intervention evaluation.

## Data Availability

The data analyzed in this study is subject to the following licenses/restrictions: All data are available from the corresponding author on reasonable request. Requests to access these datasets should be directed to Zhengxiu Luo, luozhengxiu816@hospital.cqmu.edu.cn.
