# Supplementary material for: Atelectasis predicts poor prognosis in pediatric macrolides-unresponsive Mycoplasma pneumoniae pneumonia with A2063/2064G mutations treated with azithromycin
Source: Front Cell Infect Microbiol. 2025 Jun 27;15:1604102. doi: 10.3389/fcimb.2025.1604102 (PMC12245878; doi:10.3389/fcimb.2025.1604102)
Supplement: Supplementary file 1 [file Table1.docx]

**Table S1 Adjusted logistic regression analysis for risk factors of short-term poor prognosis (RMPP) (n=82)**

| Variables | Model 1 (Unadjusted) | | | Model 2 (Model 1 plus Consolidation) | | | Model 3 (Mode 2 plus Age ) | | |
| --- | --- | --- | --- | --- | --- | --- | --- | --- | --- |
|  | OR | 95% CI | P | OR | 95% CI | P | OR | 95% CI | P |
| Atelectasis (n, %) | 4.02 | 1.03-16.00 | 0.043* | 4.04 | 1.05-16.10 | 0.042* | 4.25 | 1.07-17.67 | 0.040* |
| Consolidation (n, %) | - | - | - | 2.95 | 0.49-32.84 | 0.295 | 2.35 | 0.40-24.28 | 0.394 |
| Age (years) | - | - | - | - | - | - | 0.84 | 0.62-1.10 | 0.212 |

*****with statistical significance, P <0.05.

**Table S2 Adjusted logistic regression analysis for risk factors of long-term poor prognosis (BO or bronchiectasis diagnosed within one year after discharge) (n=82)**

| Variables | Model 1 (Unadjusted) | | | Model 2 (Model 1 plus Consolidation) | | | Model 3 (Mode 2 plus Age ) | | |
| --- | --- | --- | --- | --- | --- | --- | --- | --- | --- |
|  | OR | 95% CI | P | OR | 95% CI | P | OR | 95% CI | P |
| Atelectasis (n, %) | 5.62 | 1.04-32.80 | 0.045* | 5.61 | 1.04-32.52 | 0.044* | 5.91 | 1.07-35.65 | 0.042* |
| Consolidation (n, %) | - | - | - | 1.43 | 0.17-31.60 | 0.769 | 1.27 | 0.14-29.64 | 0.847 |
| Age (years) | - | - | - | - | - | - | 0.94 | 0.64-1.36 | 0.730 |

*****with statistical significance, P <0.05.

**
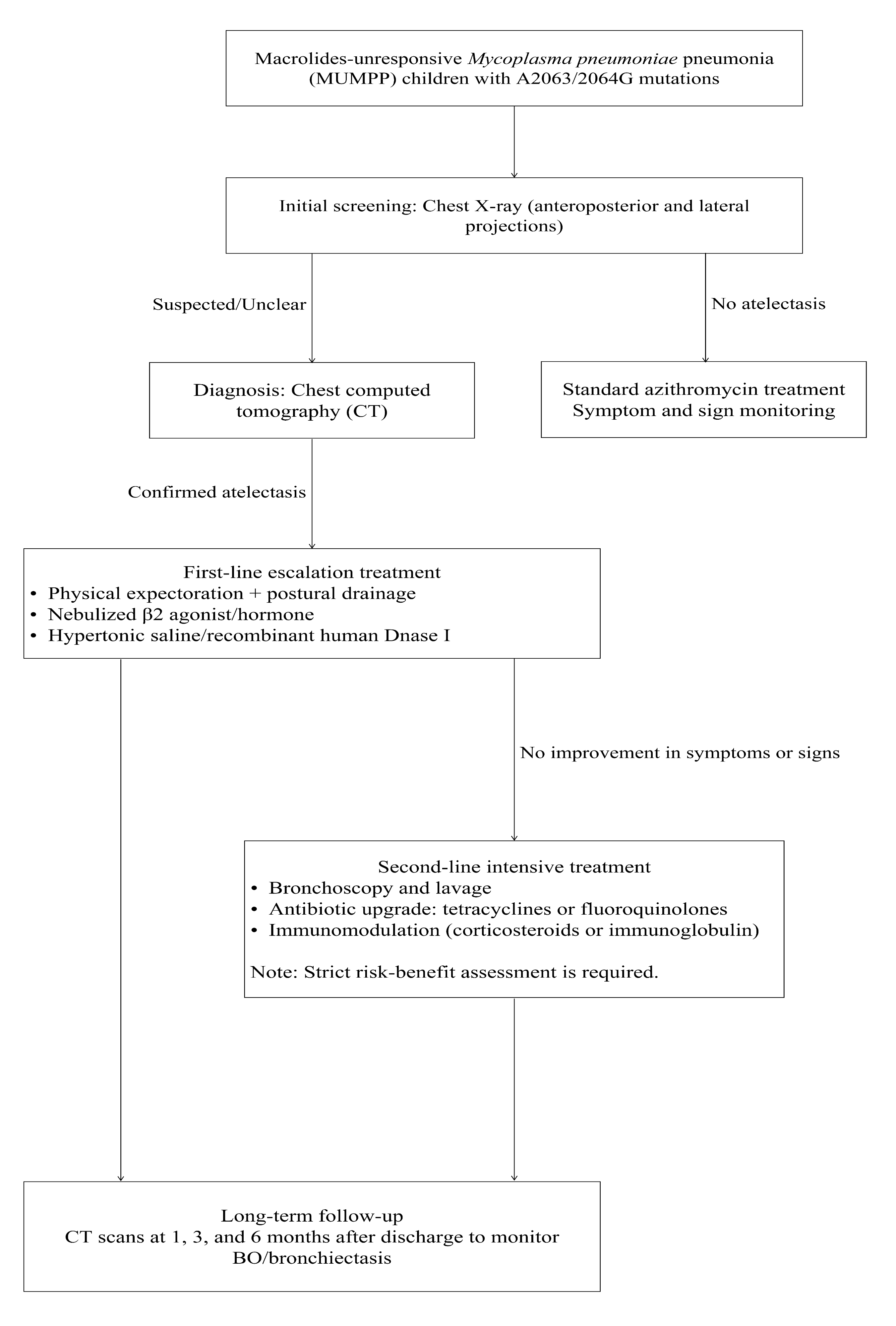
**

**Fig. S1 Clinical protocol for atelectasis management in MUMPP children with A2063/2064G mutations**
